# Sustained attention induces altered effective connectivity of the ascending thalamo-cortical relay in obsessive-compulsive disorder

**DOI:** 10.3389/fpsyt.2022.869106

**Published:** 2022-08-10

**Authors:** Mario A. Yacou, Asadur Chowdury, Philip Easter, Gregory L. Hanna, David R. Rosenberg, Vaibhav A. Diwadkar

**Affiliations:** ^1^Department of Psychiatry and Behavioral Neurosciences, Wayne State University School of Medicine, Detroit, MI, United States; ^2^Department of Psychiatry, University of Michigan, Ann Arbor, MI, United States

**Keywords:** dynamic causal modeling, obsessive-compulsive disorder, sustained attention, fMRI, thalamo-cortical relay

## Abstract

Abnormal function of the thalamo-cortical relay is considered a hallmark of obsessive-compulsive disorder (OCD) and aberrant network interactions may underpin many of the clinical and cognitive symptoms that characterize the disorder. Several statistical approaches have been applied to *in vivo* fMRI data to support the general loss of thalamo-cortical connectivity in OCD. However, (a) few studies have assessed the contextual constraints under which abnormal network interactions arise or (b) have used methods of *effective* connectivity to understand abnormal network interactions. Effective connectivity is a particularly valuable method as it describes the putative *causal* influences that brain regions exert over each other, as opposed to the largely statistical consistencies captured in functional connectivity techniques. Here, using dynamic causal modeling (DCM), we evaluated how *attention demand* induced inter-group differences (HC ≠ OCD) in effective connectivity within a motivated thalamo-cortical network. Of interest was whether these effects were observed on the *ascending* thalamo-cortical relay, essential for the sensory innervation of the cortex. fMRI time series data from sixty-two participants (OCD, 30; HC, 32) collected using an established sustained attention task were submitted to a space of 162 competing models. Across the space, models distinguished between competing hypotheses of thalamo-cortical interactions. Bayesian model selection (BMS) identified marginally differing likely generative model architectures in OCD and HC groups. Bayesian model averaging (BMA), was used to weight connectivity parameter estimates across *all models*, with each parameter weighted by each model’s posterior probability, thus providing more stable estimates of effective connectivity. Inferential statistical analyses of estimated parameters revealed two principal results: (1) Significantly reduced *intrinsic* connectivity of the V1 → SPC pathway in OCD, suggested connective weakness in the early constituents of the dorsal visual pathway; (2) More pertinent with the discovery possibilities afforded by DCM, sustained attention in OCD patients induced significantly reduced *contextual* modulation of the ascending relay from the thalamus to the prefrontal cortex. These results form an important complement to our understanding of the contextual bases of thalamo-cortical network deficits in OCD, emphasizing vulnerability of the ascending relay.

## Introduction

Obsessive-compulsive disorder (OCD) is a common neuropsychiatric disorder characterized by excessive anxiety-inducing thoughts (obsessions) that lead to repetitive anxiety-reducing behaviors (compulsions). In youth, incidence rates of OCD are high, and sub-clinical OCD symptoms are highly prevalent (1). Elucidating brain network dysfunction is of significant importance in clinical neuroscience because network neuroscience provides the best hope of identifying mechanistic pathways in the brain (2), and because the relative stability of OCD symptoms into adulthood suggests that trait-related network dysfunction may underpin this condition (3). The goal of identifying plausible mechanistic pathways has been central to all of medicine (2, 4), because an understanding of such mechanisms can result in better outcomes. Moreover, such understanding can serve as targets for assessing the efficacy of pharmacological or other interventions in OCD. This may be particularly important as many first-line treatments such as cognitive behavioral therapy (CBT) and selective serotonin reuptake inhibitors (SSRIs) remain ineffective in a substantial number of patients (5). Of particular interest is the characterization of *causal* or *directional* interactions between brain network constituents (4). Recovering causal relationships within the brain is a central challenge in neuroscience, largely because the activity of network constituents is generally not driven by single causes, and network constituents do not exert mono-causal effects on each other (6, 7). Rather, network interactions emerge from partitions of neuronal states over progressively coarser scales and that retain a measure of directionality (8). It is acknowledged that largely correlative measures for *functional* connectivity, while valuable in capturing time ordered statistical regularities between the activity of network constituents, do not provide an understanding of how network constituents impact *each other* (4). However, such interactions can be successfully recovered from fMRI times series data using dynamic causal modeling (DCM), *the* central method for *effective connectivity* analyses (9). In contra-position to functional connectivity, effective connectivity seeks to mechanistically model network interactions by recovering experimental and time-dependent, observed timing relationships between recorded neurophysiological signals (9). Thus, in the context of conditions like OCD the application of DCM *can* elucidate the functionally-evoked signatures of network dysfunction, and understanding *task-evoked* network dysfunction is key to understanding brain network dysfunction in neuropsychiatric disorders (10). Indeed, DCM has been used to study dysfunctional effective connectivity in OCD youth in domains including emotional processing (11, 12) and cognitive control (13). Herein, we provide the first extension of DCM to understand if and how task-processing during the more basic domain of sustained attention, evokes disordered effective connectivity in OCD youth (14).

Sustained attention involves the maintenance of vigilance, or tonic alertness, in the service of task persistence over sustained periods of time (15). Unsurprisingly, sustained attention is seen as a quantitative biological trait that is reliable and reasonably heritable (16), and therefore an intermediate phenotype across many psychiatric conditions including OCD (17). Impairments in sustained attention are “upstream” from other critical higher cognitive domains such as learning and memory (18). In real world settings, a loss in cognitive competence in the classroom in patients with OCD has been associated with stigmatizing attitudes amongst poorly informed teachers (19). Thus, a focus on sustained attention in OCD is not simply an issue of scientific curiosity, but one of direct clinical relevance.

Sustained attention is sub served by critical thalamo-cortical interactions (20), with the ascending thalamo-cortical relay to the dorsal prefrontal cortex (dPFC) being particularly important in mediating cortical innervation and network synchronization (21). Notably, effective connectivity of thalamo-cortical networks in healthy youth (22), reveals an intricate interplay between sustained attention processing and the impact of such processing on changes in the effective connectivity of thalamo-cortical pathways. However, such interactions have not been assessed in OCD. This is a critical lacuna because understanding altered thalamo-cortical network function in OCD *may* elucidate how the antecedents of higher-order behavioral dysfunction in OCD lies in functional impairments in more rudimentary brain pathways (23). Here we applied DCM to discover whether sustained attention evoked differences in the effective connectivity of frontal-striatal-thalamic networks in OCD youth and typical controls. Sustained attention was manipulated using a specifically tailored version of the continuous performance task (CPT) (14). The task required participants to remain vigilant for extended periods (∼120 s) during which time, numbers were presented in rapid sequence. Participants were required to detect repeated instances of numbers. A simple parametric manipulation of attention demand was evoked by changing numerical magnitude: thus, alternating epochs consisted of either only two- (low demand) or three- (high demand) digit numbers. This parametric manipulation typical of sustained attention paradigms was intended to vary the behavioral demands placed on brain networks (22, 24, 25), wherein any demand-induced changes would be reflected in estimates of effective connectivity discovered with DCM (26).

### Sustained attention, thalamo-cortical networks and relevance for obsessive-compulsive disorder

Why are thalamo-cortical networks central to sustained attention? All incoming sensory inputs (except olfactory) pass through the thalamus, and the structure has been viewed as a “relay station” and “gateway” to subcortical and cortical regions (27, 28). Anatomically, several distinct thalamic sub-nuclei synapse onto pertinent attention-related brain cortices (29, 30) including the dorsal anterior cingulate cortex (dACC), dPFC, primary visual cortex (V1), and the superior parietal cortex (SPC). In this regard, ascending relays from the thalamus assume importance.

Thalamic transmission of sensory information can be subdivided into first-order and higher-order relays. In first-order relays, thalamic nuclei convey the initial sensory inputs from sensory organs (i.e., eyes) to the cortex. For instance, retinal inputs travel to the lateral geniculate nucleus (LGN) of the thalamus before being relayed to the V1. In *higher-order relays*, thalamic nuclei receive descending inputs from cortical regions before relaying and controlling the flow of information back to the cortex (31–33). These descending (cortico-thalamic) and ascending (thalamo-cortical) pathways cumulatively underpin communication in brain networks (34), and suggest an important role for thalamic engagement and dynamic regulation of attentional, decision-making, executive control processes in the cortex (35).

Extant studies suggest that networks associated with sustained attention are subsets of the overall thalamo-cortical system (36). The reliably identified constituent regions of the attention network include frontal-striatal regions such as the dACC, the dPFC and the basal ganglia (BG)—all important for executive or supervisory mechanisms of attention (37, 38). The dACC functions as a “control center” organizing and recruiting executive brain regions like the dPFC (39–42). Additionally, the dACC shares bilateral connections to the dPFC, with the latter region known to be involved in the critical functioning of vigilance, executive control, working memory, and selective and divided attention (43). The BG are a varied group of subcortical nuclei involved in the functioning of eye movement (44), working memory (45), decision making (46), and in the organization of motivations that lead to the execution of goal-directed behaviors (e.g., organizing task-driven responses) (47). In addition to these “heteromodal” regions, multiple regions have been associated with spatial attention and orientation and sensory gating including the SPC, involved in the allocation of spatial attention, which is innervated by the V1 via the dorsal stream (48, 49) as well as direct thalamic connections (27, 40, 50).

Is there evidence of disordered brain function in OCD patients during attention processing? Utilizing a cued task-switching paradigm, Gu et al. found significantly reduced levels of brain activation in cortical (dPFC, dACC, and orbitofrontal cortex) and subcortical (caudate nucleus) regions, replicating prior studies on impaired executive functioning and cognitive flexibility (51); the task-switching paradigm required subjects to *disengage* attention from a prior task, resolve interference, and configure to a new cognitive task-set (52). Attention-related mechanisms in OCD have also been probed using the Stroop task, that measures functions related to selective attention, interference control, and cognitive flexibility (53). Exploring inhibition control in OCD, Page et al., used three different motor and cognitive control tasks (Go/No-go, motor Stroop, Switch tasks) to assess dysfunctional brain *activations*. The authors found reduced activation in OCD in multiple cortical (DLPFC, OFC), striatal, and thalamic regions (54). In a motor task with substantial attention demands, Meram and colleagues demonstrated disordered network-based mechanisms of frontal control in OCD (55), a pattern that replicates prior studies using working memory (56). Finally, Woolley et al. used an inhibitory (‘stop’) task, to show reduced activation of frontal, striatal and thalamic regions in pediatric OCD, evidence of dysregulation of fronto-striatal-thalamic (FST) regions during motor inhibition (57). Given that thalamic inputs are highly salient in driving cortical networks, and given the loss of cortical network tone in OCD, Del Casale and colleagues have suggested that altered thalamic gating in OCD, results in alterations in cortical activity in the disorder, leading to the subsequent emergence of core clinical characteristics including intrusive thoughts and repetitive and ritualistic behaviors (58). Is there any evidence of altered effective connectivity of ascending and/or descending relays of the thalamo-cortical network in OCD using *task*-based fMRI? To our knowledge this specific issue has not been addressed with DCM.

### Functional connectivity studies in obsessive-compulsive disorder

What insights in OCD have we gained from functional connectivity studies? In the past 20 years, task-positive and resting-state functional connectivity studies have expanded our understanding of brain circuit abnormalities in OCD (59). Chiefly, our understanding of dysfunctional cortical, striatal, and thalamic (CSTC) circuits and resting-state networks [i.e., default-mode network (DMN) circuits] has been greatly expanded. Task-based fMRI studies have revealed abnormal patterns of hypo- and hyper-activation in domains such as executive functioning, emotional processing, and symptom provocation (59) with commonly implicated brain regions including: the prefrontal cortex (PFC) (54, 60), BG and thalamus (61), orbitofrontal cortex (OFC) (62), dACC (63), amygdala (64).

What is effective connectivity and how can it expand our understanding of dysfunctional brain networks in OCD? While functional connectivity describes statistical dependencies between times series, effective connectivity rests on a mechanistic model of the neurophysiological processes that *generated* the *observed* data (65). Thus, it is plausible to arrive at an understanding of the casual interactions one neuronal unit exerts over another; the elucidation of these causal influences via effective connectivity studies may prove pivotal in the characterization of aberrant pathways in OCD. Indeed, attempts to understand *directed* connectivity have been conducted using approaches such as psychophysiological interaction (PPI) (66) or structural equation modeling (SEM) (67). However, these approaches rely on *observed* fMRI signals (65). We relied on DCM as we were specifically motivated to recover causal effects between brain regions in OCD patients and controls.

### Effective connectivity using dynamic causal modeling

Dynamic causal modeling allows for the interpretation of *causal* interactions between hidden state variables (9, 26) by modeling the brain as a bilinear input-output system. In this framework, inputs to the system are the experimental conditions and stimuli, and the outputs from the system are fMRI measured hemodynamic responses. DCM evaluates and assesses competing neurobiologically plausible models to determine any model’s posterior evidence for predicting the observed fMRI data. The competing models constitute a motivated *a priori* hypothesis space. Posterior probabilities are used to weight parameter estimates and differences between estimated parameters can be investigated using standard inferential statistics (26, 68). The following state differential equation is implemented to measure changes in neural responses:


$$\frac{dx}{dt}=\left(A+\sum_{j=1}^{m}u_{j}B^{\left(j\right)}\right)x+Cu$$

Where, “A” represents the task-independent intrinsic (endogenous) coupling that exists between brain regions of interest, “B^(j)^” represents the task-dependent modulation of intrinsic connections via experimental manipulations, and variable “C” represents the sensorimotor driving inputs on cortical regions. We surmise that characterizing ascending thalamic-cortical relays in OCD is essential and that DCM applied to fMRI data collected during sustained attention, provides that ideal combination of method *and* task (4, 22, 69). Therefore, DCM allowed us to specifically assess the effects of task conditions on the ascending and descending relay and how (and why) any contextual modulation on thalamo-cortical relays might differ in OCD participants relative to controls.

## Materials and methods

### Subjects (participants)

Thirty OCD subjects (Age: 10.1–21.9 years; Mean Age: 16.2 years.; 10 males) and 32 healthy controls (Age: 10.9–21.1 years; Mean Age: 16.7 years; 12 males) provided informed consent or assent to participate in the fMRI study. For assenting participants, parental consent was also obtained. Participants’ IQ (89 < IQ < 143; Mean: 109.8; SD: 11.3) was assessed using either the Wechsler Intelligence Scale for Children (WISC) or the Wechsler Adult Intelligence Scale (WAIS). Healthy controls were evaluated for, and cleared of, any psychiatric illness. OCD participants were evaluated by a psychiatrist. Demographic and clinical characteristics of study participants can be viewed in Table 1.

**TABLE 1:** Demographic and clinical characteristics.

Characteristic	OCD (*n* = 30)	HC (*n* = 32)
Sex (M/F)	10/20	12/20
*Age*		
Mean (SD)	16.2 (3.3)	16.7 (2.8)
Range	10.1–21.9	10.9–21.1
*CY-BOCS*		
Lifetime obsessions (SD)	14.3 (4.6)	–
Lifetime compulsions (SD)	13.7(4.0)	–
Lifetime total (SD)	28.1(7.5)	–

OCD, obsessive-compulsive disorder; HC, healthy controls; SD, standard deviation; CY-BOCS, Children’s Yale-Brown Obsessive-Compulsive Scale.

The Schedule for Obsessive-Compulsive and Other Behavioral Syndromes and Schedule for Schizophrenia and Affective Disorders for School-Aged Children-Present and Lifetime Version were used to interview participants and their parents (70, 71). Furthermore, a modified version of the Children’s Yale-Brown Obsessive-Compulsive Disorder Scale (CY-BOCS) was used to measure the lifetime (maximum) and current severity of OCD participants (72, 73). Using DSM-5 criteria, clinicians independently confirmed the lifetime and current Axis I diagnosis. Participants with the following medical or psychiatric criteria were excluded: lifetime history of schizophrenia, bipolar disorder, substance abuse/dependence, anorexia nervosa, bulimia nervosa, epilepsy, head injuries resulting in chronic loss of consciousness, Huntington’s disease, dyskinesia, autism, IQ ≤ 80 or ≥ 15 on the lifetime version of the Social Communication Questionnaire (74, 75). The Wayne State University School of Medicine (WSUSOM) and the UM Human Investigation Committee approved our study.

### fMRI task and data collection

**FIGURE 1:**
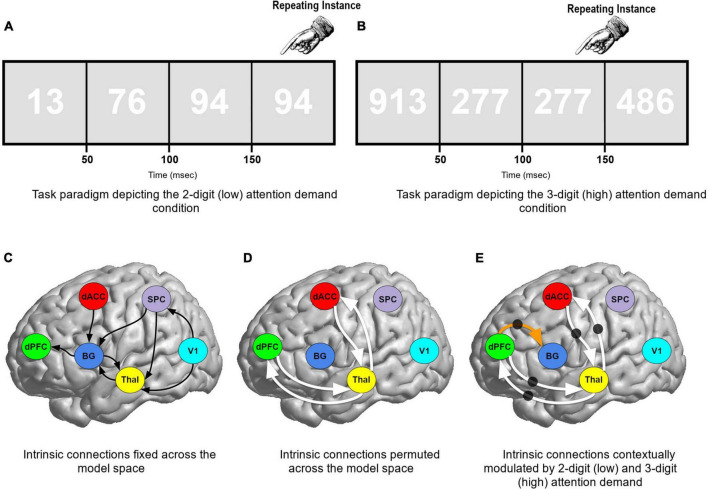
**(A,B)** The paradigm employed and the low and high demand conditions are schematically depicted. Participants performed a continuous performance task, identical pairs version (CPT-IP), and identified repeating instances of either 2- or 3-digit numbers (blocked in 120 s epochs). The magnitude of targets constitutes a parametric manipulation of attention demands: low (2-digit) or high (3-digit) attention demand. The remainder of the figure reveals the model space used for DCM. **(C)** Solid (curved) black lines represent the intrinsic connections fixed across the model space. **(D)** Solid white lines represent cortical-thalamic intrinsic connections that were permuted across the model space. The black circles **(E)** denote connections which were contextually modulated by both low (2-digit) and high (3-digit) attention demand conditions. Thus, the solid white lines were permuted for both intrinsic connectivity AND contextual modulation. The dPFC → BG connection (orange arrow) was contextually modulated when the intrinsic connection was present.

All participants were administered a modified version of the Continuous Performance Task, Identical Pairs (CPT-IP) (Figure 1) deployed in previous studies (14, 22, 76), and required subjects to detect *repeated* numbers within a rapidly presented sequence. In separate blocks, two- or three-digit numbers were presented (50 ms, 250 ms SOA) over extended block lengths (120 s). The extended blocks were specifically designed to induce *sustained* (as opposed to transient) attention. Titrating demand in sustained attention has typically been implemented by changing the onsets between successively presented stimuli (77) or by experimentally manipulating stimulus characteristics (as undertaken here). Previous studies that have analyzed behavioral performance have demonstrated that the numerosity of the targets does evoke a lucid parameterization of task difficulty associated with attention-demand (Low vs. High); detection sensitivity (*d’*) for targets (repeated numbers) during three-digit epochs is lower than detection sensitivity for targets during two-digit epochs (14). Thus, attention demand constitutes a potentially critical modulator of the effective connectivity of ascending and/or descending thalamo-cortical relays (22, 78). In addition, 20 s rest epochs interspersed the experiment allowing participants some recovery between task-active epochs.

480 stimuli were shown in each block (25% were targets). To diminish figure-ground contrast (and increase attention commitments), numbers were presented in white numerical text (RGB: 255,255,255) against a grey background (RGB: 225,225,225) (79). Furthermore, to prevent selection of consecutive digits based on low-level features such as the lack of a flicker between repeated numbers, participants were presented with alternating fonts for successive digits in the sequence (78) thus ensuring a constant level of flicker throughout each task-active epoch.

### Data acquisition

Gradient echo echo-planar imaging (EPI) fMRI data were obtained using a 3 Tesla Siemens Verio system (12-channel volume coil head) at the Vaitkevicius Magnetic Resonance Center. The following parameters were used for the T_2_* fMRI acquisition: TR: 2.6 s, echo time; TE: 29 ms; matrix dimensions: 128 × 128; voxel dimensions: 2 mm × 2 mm × 2 mm; field of view (FOV): 256 mm × 256 mm; 36 axial slices. The 3D matrix (128 × 128 × 36) provided very high resolution coverage of the cortex for a conventional EPI T_2_* sequence. The 36 axial slices were positioned parallel to the anterior commissure/posterior commissure (AC-PC) line and provided comprehensive coverage of the cerebrum. A T_1_-weighted structural image was obtained for preprocessing and co-registration using a 3D Magnetization Prepared Rapid Gradient Echo (MPRAGE) sequence with the following parameters: TR: 2,200 ms; TI: 778 ms; TE: 3 ms; FOV: 256 mm × 256 mm; matrix dimensions: 256 × 256; flip-angle: 13°; 256 axial slices, 1.0 mm thickness. All scans were reviewed by a neuroradiologist to exclude clinically significant irregularities.

### fMRI processing (image preprocessing)

Image processing was undertaken in SPM12^1^ using established methods for temporal (slice timing correction) and spatial preprocessing. First, EPI images were manually oriented to the AC-PC line with the reorientation vector applied across the EPI image set, realigned to a reference image to correct for head movement, and co-registered to the anatomical high resolution T_1_ image. The T_1_ image was normalized to the Montreal Neurological Institute (MNI) template, with the resultant deformations applied to the co-registered EPI images. Low frequency components were removed with a lenient filter (1/256 as opposed to 1/128) and this filter accommodated longer block lengths (120 s) that were used to induce sustained, as opposed to transient attention (14, 22). Images were resliced to (2 mm^3^) and smoothed (8 mm FWHM). At the first level, epochs were modeled with boxcar stimulus functions convolved with a canonical hemodynamic response function to form regressors of interest, with the six motion parameters (3 for translation and 3 for rotation) from the co-registration modeled as covariates of no interest. We did not model phasic or event-related responses to targets because we were interested in the responses specifically associated with *sustained* attention. Images exceeding 4 mm of movement (<1% of all images) were excised from analyses. This approach to image processing and modeling is consistent with recent publications (55, 68, 80–84).

### Defining the dynamic causal modeling model space

**FIGURE 2:**
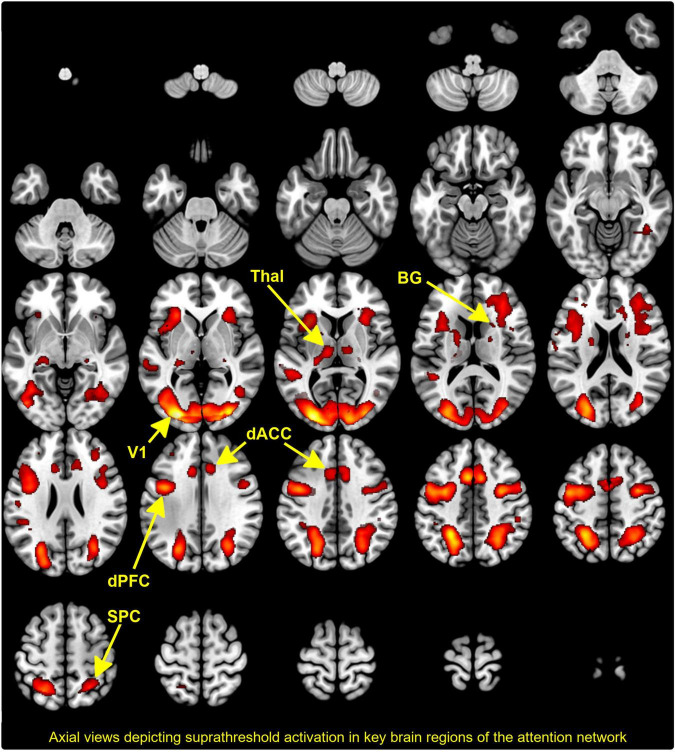
The activation map depicts the results of conjunction analyses across group and condition with co-activated clusters projected to a mosaic of axial views. Supra threshold clusters are clearly seen in the thalamus, basal ganglia (BG), dorsal anterior cingulate cortex (dACC), dorsal prefrontal cortex (dPFC), superior parietal cortex (SPC), and visual cortex (V1). As we note, these sites are consistent with previously published evidence of the engagement of the attention network.

The regressors of interest representing each of the levels of demand (Low:2-digit; High:3-digit) were submitted to a second-level mixed random effects model (85), with condition and group respectively modeled as repeated and independent factors. To identify an appropriate conjunction of co-activated clusters across groups and conditions, we used Nichols et al. version of the minimum-inference statistic (86)(minimum statistic compared to the conjunction null). These co-activations (depicted in Figure 2) conform to activation profiles identified in previous studies using identical and related paradigms (14, 22, 87). Clearly identifiable peaks are noted in regions including the V1, SPC, thalamus, BG, dACC, and the dPFC. These regions of interest provided a thalamo-cortical network of interest wherein inter-group differences in effective connectivity could be investigated without the confound of activation-differences between groups (41).

A total of 162 competing network models were configured in the hypothesis/model space. Hypotheses of interest heavily focused on evaluating the contextual modulation of specific pathways during sustained attention with varying levels of demand. Across models eight hypothesis-neutral intrinsic connections were fixed, depicted in Figure 1C by a solid black (curved) line. Four cortical-thalamic intrinsic connections were permuted across the model space represented by solid white lines (Figure 1D): (1) Thal → dPFC; (2) dPFC → Thal; (3) Thal → dACC; and (4) dACC → Thal. We tested whether these four cortical-thalamic connections when present, were contextually modulated by the task (represented by black circles in Figure 1E). Finally, the dPFC → BG connection was permuted across models and when present we tested if it was contextually modulated by the task. This 162-model space was therefore a combination of (a) three possible contextual modulation effects on four permuted intrinsic connections plus, (b) two possible effects on the dPFC → BG connection (3^4^ × 2 = 162 models).

### Model estimation

**FIGURE 3:**
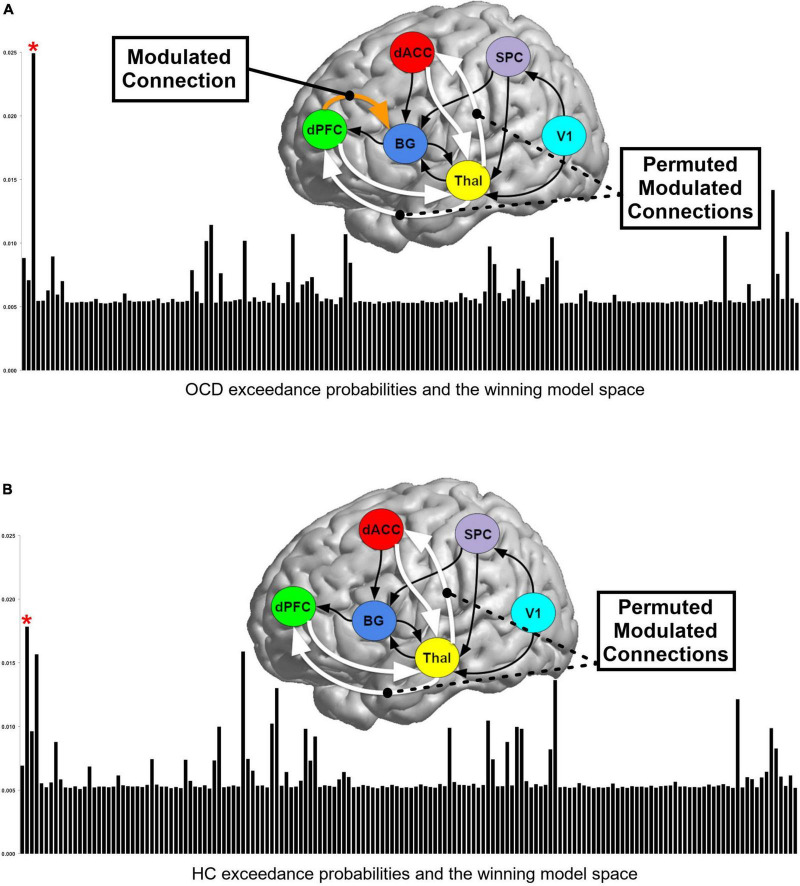
Bayesian model selection (BMS) results for (A) OCD and (B) HC. Exceedance probabilities (relative likelihood) for each of the 162 models generated are depicted in the skyscraper plots for each of the **(A)** OCD and **(B)** HC groups. Red asterisks denote models with the highest exceedance probability. BMS revealed slightly different winning models for OCD and HC groups and these differing model structures are depicted above each plot (see text for discussion).

Across each the 62 participants, time series were extracted using spheres (5 mm radius) centered on the peak of the “effects of interest” F-contrast (*p* < 0.05, adjusted for “effects of no interest”) within each region. These time series were submitted to model estimation, and the cumulative estimated model space (10,044 models, i.e., 162 models across 62 participants) was then submitted to random effects (RFX) Bayesian model selection (BMS), with an initial goal of determining the most likely generative model in each of the OCD and HC groups (Figure 3). A variational Bayes method using the RFX procedure was employed to estimate the posterior probabilities of competing models, resulting in posterior likelihoods associated with each model in the space in each group. RFX is an ideal approach for identifying model likelihood under conditions of sensorimotor or cognitive tasks (14, 26, 41).

Parameter estimation (for values associated with intrinsic connectivity and contextual modulation) was conducted using Bayesian model averaging (BMA) which generates a weighted average of the parameter estimates of each model within a group, where the weight is determined by the posterior probability of each individual model. BMA is well-optimized for reliably estimating parameter values in relatively large model spaces (88), and in a human *psychological* context has been hypothesized as being a generally rational approach to decision making (89).

## Results

**FIGURE 4:**
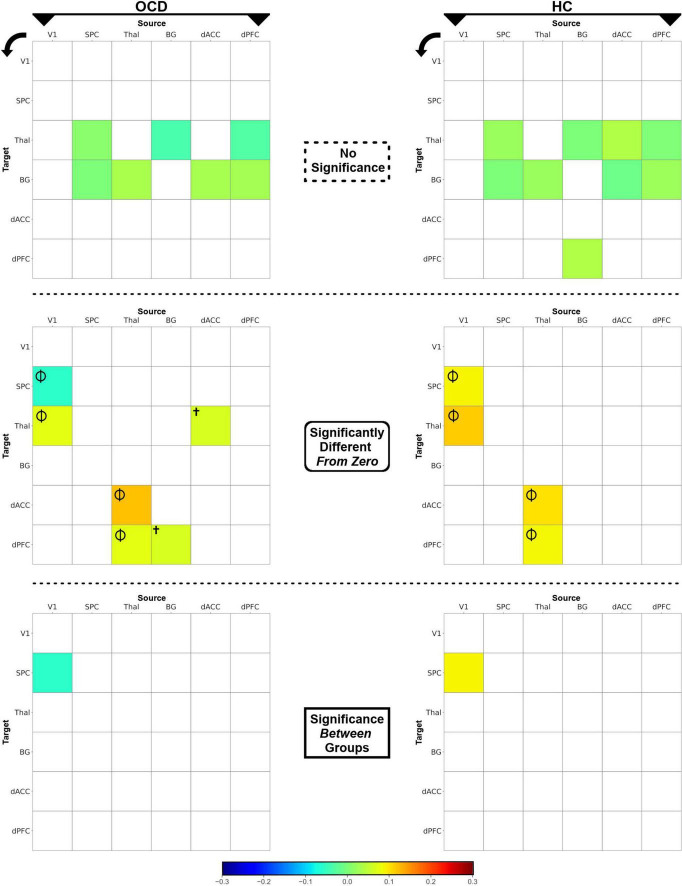
The heat maps depict posterior intrinsic connectivity means for OCD **(left)** and HC **(right)** groups. The posterior means are displayed using a blue-to-red color bar and the maps are divided by the nature of the effects (non-significant, different from zero, different between groups. **(Top row)** Non-significant posterior means are depicted for each of the OCD **(left)** and HC **(right)** groups. **(Middle row)** Posterior means which were significantly different from zero (*p* < 0.05, one-sample *t*-tests, i.e., there was significant intrinsic connectivity) are depicted. Effects observed in both groups (Φ) or only in OCD (^†^) are marked. **(Bottom row)** The intrinsic connectivity of the V1 → SPC pathway was significantly reduced in OCD, revealed in an independent-samples *t*-test.

**FIGURE 5:**
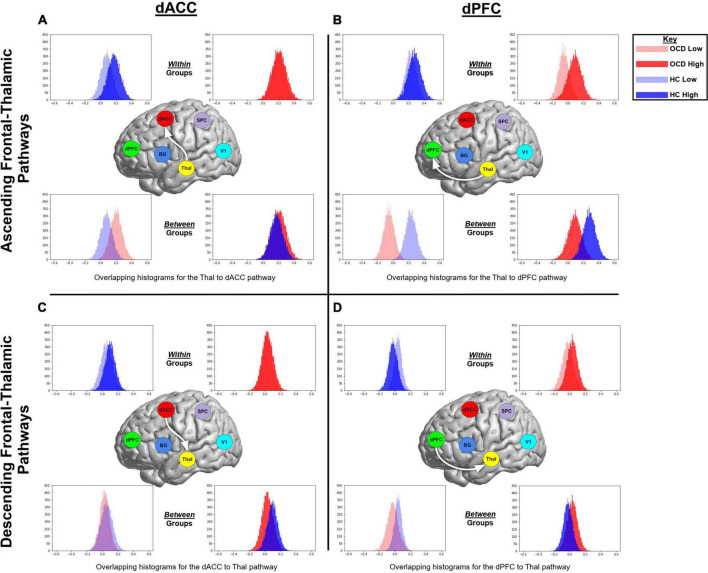
The histograms were generated from extracting 20,000 BMA samples in Occam’s window, and highlight speculative trends associated with within *or* between group parameter estimates of contextual modulation. Data from OCD are represented in red, while data from HC are in blue. Data from the more demanding attention condition are represented by opaque colors, while data from the less demanding condition in transparent colors. The figure represents ascending and descending fronto-thalamic pathways and is organized in four panels: each column represents pathways involving either the dACC or the dPFC, and each row represents the ascending (from the thalamus) or descending (to the thalamus) pathway. In each panel, histograms are presented twice; initially to depict *within* group trends in each of the task conditions **(top row)** and then to depict *between* group trends in each of the conditions **(bottom row)**.

**FIGURE 6:**
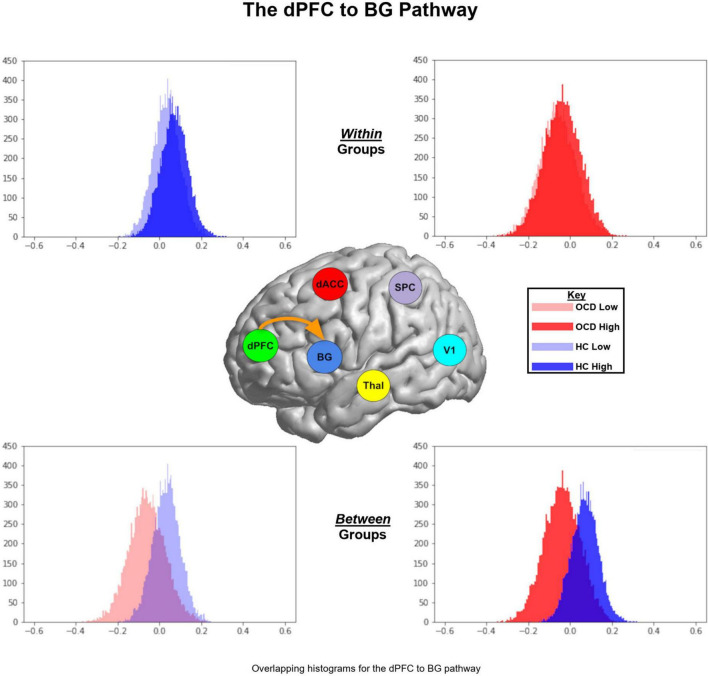
Parameter histograms for the dPFC → BG pathway are presented in similar format as Figure 5. For *both* low and high levels of demand, the parameter histograms for the HC group are shifted to the right of the OCD group.

**FIGURE 7:**
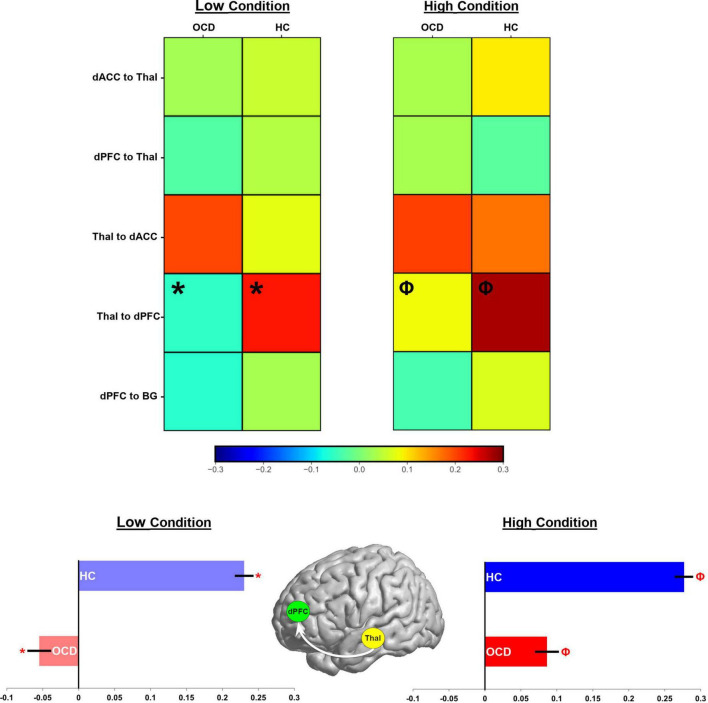
Parameter estimates for the contextual modulation of network pathways are present in heat maps (see color bar for values). Values for the low demand condition are on the left, while those for the high demand condition are on the right. In each map, groups are presented in separate columns (OCD, HC). Independent samples *t*-tests revealed statistically significant differences between OCD and HC in the Thal → dPFC pathway for both task conditions (Low demand: *; High Demand: Φ). These data are depicted in bar graphs below to accentuate the effect (±sem).

We organize our results as follows: (1) We first present exceedance probabilities across the 162-model space observed for each of the OCD and HC groups (Figure 3); (2) Heat maps are used to depict intrinsic connectivity values (following BMA) for pathways in each of the OCD and HC groups (Figure 4); (3) In Figures 5, 6, we provide histograms of parameters (generated from multiple simulations) associated with the contextual modulation of multiple pathways by each level of attention demand. These distributions motivate speculative inferences about within and inter-group differences in parameter estimates; (4) These speculations are codified in Figure 7 where we identify statistically significant inter-group differences in the contextual modulation of the ascending relay (thalamus during each of the low and high demand task conditions).

### Model selection in DCM

A perusal of the exceedance probabilities (relative likelihood) for each of the 162 competing models in Figure 3 is illustrative. In each group, no single model was characterized by substantially larger likelihood relative to its competitors and the likeliest generative models in the OCD and the HC groups differed slightly. This difference suggests that the effective network structure of task implementation in OCD differed from HC, but the relative proximity of posterior likelihoods motivate the subsequent reliance on BMA for inference (26).

### Statistical assessment of intrinsic connectivity parameters

The statistical significance of intrinsic connectivity parameters was comprehensively evaluated using a combination of one-sample *t*-tests (to identify pathways where parameters in either group were significantly different from zero), and independent samples *t*-tests (to identify pathways with significant inter-group differences in intrinsic connectivity). For ease of access, the single full matrix in each group is decomposed into a) no significant effects (top row), significantly different (from zero) connectivity parameters (middle row) and significant inter-group differences (bottom row) in Figure 4. Each asymmetric connectivity matrix represents intrinsic connectivity parameters from a source region (column) to a target region (row) with the colors calibrated by the estimated mean value (see color bar). One-sample *t*-tests indicated that in *both* OCD and HC groups, four intrinsic connectivity parameters were significantly different from zero: (1) V1 → Thal, (2) Thal → dACC, (3) Thal → dPFC, and (4) V1 → SPC (Φ in Figure 4). Two other intrinsic connectivity parameters were also significantly different from zero, but only in the OCD group: (1) BG → dPFC and (2) dACC → Thal (†in Figure 4). Finally, the results of the independent samples *t*-tests revealed significant differences in the parameters for the V1 → SPC pathway, indicating that V1 → SPC connectivity was lower in OCD. Statistical information is provided in Table 2.

**TABLE 2:** Intrinsic connectivity parameters and statistical analyses (significant effects in bold).

	HC (*n* = 32)			OCD (*n* = 30)			Inter-group
Pathway	Mean	SD	*t-score* (≠ 0)	Mean	SD	*t*-score (≠ 0)	*t*-score (OCD ≠ HC)
V1 → SPC	**0.09**	**0.12**	**4.28**	**−0.06**	**0.12**	**−2.61**	**4.83**
V1 → Thal	**0.12**	**0.12**	**5.47**	**0.08**	**0.12**	**3.33**	1.26
SPC → Thal	0.02	0.14	0.91	0.01	0.14	0.43	0.31
SPC → BG	0.00	0.02	0.00	0.00	0.02	0.00	0.00
Thal → BG	0.02	0.14	0.94	0.03	0.14	1.28	–0.28
Thal → dACC	**0.10**	**0.14**	**4.24**	**0.12**	**0.14**	**4.71**	–0.60
Thal → dPFC	**0.09**	**0.14**	**3.58**	**0.08**	**0.14**	**3.01**	0.34
BG → Thal	0.00	0.14	0.04	–0.04	0.14	–1.45	1.07
BG → dPFC	0.04	0.15	1.49	**0.07**	**0.14**	**2.52**	–0.75
dACC → Thal	0.04	0.13	1.69	0.07	0.13	2.82	–0.88
dACC → BG	–0.01	0.14	–0.43	**0.03**	**0.14**	**1.23**	–1.19
dPFC → Thal	0.00	0.13	0.18	–0.03	0.13	–1.23	1.02
dPFC → BG	0.02	0.13	1.04	0.03	0.14	1.16	–0.16

V1, primary visual cortex; SPC, superior parietal cortex; Thal, thalamus; BG, basal ganglia; dACC, dorsal anterior cingulate cortex; dPFC, dorsal prefrontal cortex. Mean, standard deviation (SD), and one sample *t*-test (t-score) are displayed for healthy controls (HC) and obsessive-compulsive disorder (OCD) subjects. The inter-group column displays t-scores from independent samples *t*-tests. Bold indicates significant differences (from zero or between groups).

### Statistical assessment of contextual modulation parameters

BMA was used to weight parameters over the 162 generative model architectures based on the posterior probability of models across a group. Thus, the likelihood of each hypothesis is represented in a quantitative estimate of the parameters for variables associated with that hypothesis. For an initial assessment, histograms were generated from extracting 2 × 10^4^ BMA samples in Occam’s window. These were used to highlight speculative trends associated with within *or* between group parameter estimates of contextual modulation of the relevant pathways by each of the attention conditions. These histograms are presented in Figures 5, 6. In each figure, data from the OCD group are represented in red, while data from the HC group are in blue. Data from the more demanding attention condition are represented by opaque colors, while data from the less demanding condition in transparent colors. Figure 5 represents ascending and descending fronto-thalamic pathways and is organized in four panels: each column represents pathways involving either the dACC or the dPFC, and each row represents the ascending (from the thalamus) or descending (to the thalamus) pathway. In each panel, histograms are presented twice; initially to depict *within* group trends in each of the task conditions (top row) and then to depict *between* group trends in each of the conditions (bottom row).

Figure 5A (top left panel) depicts two interesting trends associated with the ascending Thal → dACC pathway: (1) As seen, within the HC (but not OCD) group, the parameter histogram associated with *high* demand is shifted to the right. This suggests that in healthy controls, an *increase* in attention demand effects an *increase* in connectivity of this ascending pathway; (2) Between groups, the histogram for HC is shifted to the left of the OCD group. This effect is a between-group extension of the previously noted within-group effect. In Figure 5B (top right panel), two interesting trends characterize the Thal → dPFC pathway: (1) Within OCD, the histogram of values for the *high* demand attention condition is shifted to the right; (2) When inspecting data between groups, we observe that for *both* the low and the high demand conditions, the parameter histograms in HC are shifted to the right of those in OCD. This effect suggests that there is a general increase in the contextual modulation of this ascending relay in HC compared to OCD (or a decrease in OCD compared to HC). In Figure 5C (bottom left), the histograms for the dACC → Thal descending relay suggests that the *high* demand condition induces an overall larger, positive contextual modulation in the sample means in HC. Finally, in Figure 5D (bottom right panel), parameter histograms for the dPFC → Thal descending relay suggest that for the *low* demand condition, the parameter histogram for HC is shifted to the right of that for the OCD group. However, for the *high* demand condition, we observed a reversal of this effect. Figure 6 depicts parameter histograms for the dPFC → BG pathway revealing that for *both* low and high levels of demand, the parameter histograms for the HC group are shifted to the right of the OCD group.

The effects depicted in Figures 5, 6 are suggestive, but motivated more robust parametric analyses in the service of inference. Accordingly, parameter estimates across participants and groups were submitted to independent sample *t*-tests to identify pathways with significant inter-group differences. Figure 7 (top) uses a heat map to visualize parameter means in each of the OCD and HC groups. Data are separated into each of the low and high demand conditions. Cells marked by symbols constitute a member of a pair with significant differences in contextual modulation (see Table 3 for details). Two effects were significant, both observed on the ascending Thal → dPFC relay. Both the low and the high attention demand conditions induced greater contextual modulation in HC compared to OCD. These significant effects were pulled from the heat maps with the group means visualized in the bar graphs in Figure 7 (bottom) (±sem).

**TABLE 3:** Parameters for contextual modulation and statistical analyses (significant effects in bold).

	HC (*n* = 32)		OCD (*n* = 30)		
Pathway	Mean	SD	Mean	SD	*t*-score (OCD ≠ HC)
*Low demand condition*					
dACC → Thal	0.05	0.28	0.03	0.25	–0.39
dPFC → Thal	0.04	0.22	–0.03	0.27	–1.12
Thal → dACC	0.08	0.28	0.20	0.28	1.74
**Thal → dPFC**	**0.23**	**0**.**27**	**−0.06**	**0**.**27**	**−4.21**
dPFC → BG	0.03	0.25	–0.06	0.30	–1.32
*High demand condition*					
dACC → Thal	0.10	0.26	0.04	0.27	–0.90
dPFC → Thal	–0.02	0.24	0.03	0.26	0.88
Thal → dACC	0.17	0.29	0.21	0.31	0.43
**Thal → dPFC**	**0.28**	**0**.**29**	**0.09**	**0**.**30**	**−2.54**
dPFC → BG	0.07	0.26	–0.04	0.30	–1.54

Thal, thalamus; BG, basal ganglia; dACC, dorsal anterior cingulate cortex; dPFC, dorsal prefrontal cortex. Mean, standard deviation (SD), and independent samples *t*-test (t-scores) are displayed for healthy controls (HC) and obsessive-compulsive disorder (OCD) subjects for the low and high attention demand conditions. Bold indicates significance between groups. *p* < 0.05.

## Discussion

In this study, we utilized DCM to assess the impact of sustained attention at varying levels of demand on the effective connectivity of cortical-striatal-thalamic pathways in OCD and HC. The DCM investigations were underpinned by a complex hypothesis-driven model space with 162 competing models/hypotheses. Across the space, we evaluated (a) the likelihood of specific intrinsic connections being present, and (b) whether task conditions modulated the connectivity of ascending and descending frontal-thalamic pathways. Bayesian model selection (BMS) was employed to sift between the predictive models for each group, with a focus of the analyses relying on Bayesian model averaging (BMA) to evaluate group differences in weighted posterior parameter estimates. This approach provides a more inclusive, albeit weighted approach toward the contributions of the different models to our results (88).

From these investigations, we discuss two principal results: (1) Analyses of parameter estimates revealed that the V1 → SPC pathway showed significantly weaker intrinsic connectivity in OCD (Figure 4, bottom row); (2) Most compellingly, task conditions during sustained attention evoked statistically significant differences (OCD < HC) for the ascending Thal → dPFC relay (Figure 7), a central pathway through which thalamic inputs innervate the cortex (90). These highly circumscribed results reaffirm the contextual bases of sensorimotor processes in brain network function (6, 91). Moreover, they motivate two sets of inferences that are related to relatively early sensory and visual pathways. The first is that in OCD, contextual modulation of the ascending Thal → dPFC pathway during sustained attention is greatly reduced. This constitutes evidence for task-induced alterations in the functionality of this ascending relay. This deficit is observed even as contextual modulation of other pathways across the CST connective circuitry remained largely intact. Secondly, portions of the dorsal visual pathway show reduced *intrinsic* connectivity in OCD. This reduction is tentative evidence in support of the hypothesis that early sensory processing might be impaired in OCD (92, 93). The approach and results highlight the value of DCM in the discovery of intact and dysfunctional network structure (94), and our observations of functional loss provide previously unknown insights on how behavioral context evokes circumscribed pathology in the OCD brain. In the remainder of the paper, we discuss the putative import of the observed effects and their relevance for our understanding of the brain network expressions of OCD.

### A loss of intrinsic connectivity of the dorsal pathway and its relevance for obsessive-compulsive disorder

The dorsal visual pathway has long been presumed to play a key role in the transmission of spatial aspects of visual processing in the service of higher sensori-motor function (49). The V1 receives visual sensory information via the lateral geniculate nucleus (LGN) of the thalamus before sending projections to subsequent brain regions (95). Likewise, the SPC is also innervated by thalamic connections (27, 40, 50) and functions in the allocation of spatial attention. How does visual information interface between occipital and parietal cortices? As noted, current understanding rests on the two-streams (dorsal and ventral) as being the purported mechanism of action (96). The dorsal visual stream (i.e., V1→ SPC) functions in computing spatial relationships and orienting of visuospatial attention, often referred to as the “where” pathway (48, 97, 98). In contrast, the ventral visual stream functions in the recognition of objects and faces, often described as the “what” pathway (48, 97, 98).

Evidence from *in vivo* MRI studies tends to be consistent with these theoretical motivations. Resting state functional connectivity studies have shown that connectivity between the V1 and superior parietal lobe (SPL) is predictive of visual search efficiency which relies heavily on the spatial components of vision (99). Thus, visual search tasks induce greater engagement of brain regions that are part of the dorsal visual stream including the occipital cortex, the SPL and occipital cortex, and beyond including the dACC, and inferior frontal cortex. Moreover, participants who are more effective in visual search show stronger functional connectivity between the SPL and visual cortex. Thus, a finding of weaker *intrinsic* connectivity of the V1 → SPC pathway implies that in OCD there may be reduced ability of the dorsal visual stream to effectively transmit spatial information from the V1. Is there evidence for visuo-spatial processing deficits in OCD?

Indeed, existing studies have frequently described problems with visuo-spatial processing in OCD patients (100, 101). For example, poor fine-motor and visuospatial skills in pediatric OCD appear to predict the presence of the disorder well into adulthood (102). In fact, deficits in visual memory, especially when complex visual stimuli are involved, is one of the most commonly reported dysfunctional cognitive domains in OCD (103–105). One meta-analysis of cognitive functioning in OCD showed that OCD patients experience impairments in visuospatial memory, visual organizational skill, and executive functions such as planning ability (105). Other studies on OCD have also shown deficits in spatial working memory and verbal/nonverbal recall (104, 106, 107). A few early studies on youth with Tourette’s syndrome and/or ADHD disorders revealed slowed reaction times on continuous performance tasks, implying a reduced ability of subjects to remain vigilant compared to controls (108, 109). Employing numerous neurocognitive tasks (e.g., Finger Windows task), Chang et al showed spatial attention deficits in OCD youth (110); this result was consistent with prior adult OCD studies on memory, spatial attention, and other executive functions (106, 111, 112). Our observations of reduced intrinsic connectivity of the V1→ SPC are indeed consistent with these results.

### A loss of contextual modulation of the ascending relay in obsessive-compulsive disorder

Sensory inputs and outputs to the thalamic nuclei can be subdivided into first-order relays and higher-order relays. In first-order relays, thalamic nuclei receive input from sensory organs (i.e., the eye) before relaying information to the cortex. For instance, visual information from the retina is relayed from the LGN of thalamus to the V1 before further processing of visual information can occur. In higher-order relays, thalamic nuclei receive descending inputs from cortical regions before relaying and controlling the flow of information back to the cortex, forming cortico-thalamo-cortical pathways (31–33). Studies on the lateral pulvinar nucleus’ role in cortical processing of visual information further supports the expanded modulatory role of some thalamic nuclei; removing lateral pulvinar input to the V1 nearly extinguishes “visual responses in the primary visual cortex,” suggesting that the pulvinar nucleus plays a critical and integral role in the functioning of the visual cortex (31). Due to relaying more “processed” inputs flowing back to the cortex, higher-order relays have been viewed as taking a more active and modulatory role in routing inputs (113).

Because groups of thalamic nuclei are involved in relaying and controlling the flow of sensory and motor information to the cortex, the thalamus has been dubbed the “relay station” or “gatekeeper” of cortical-bound information (27, 28, 114, 115). Thalamic inputs to the dPFC assume particular importance, given this heteromodal structure’s role in a multiplicity of processing domains including working memory, selective, divided, and sustained attention, and executive function (43, 116). Far from being a passive relay in the bottom-up gating of attentional (and other sensory) information to the cortex, the thalamus is now thought to exert more constructive influences on the dynamic upward flow of sensory information. In addition to a vast array of cellular studies (117–120), this framework has also been confirmed by DCM studies showing that ascending relays from the thalamus to the dPFC are contextually modulated by sustained attention (22). Such DCM based studies affirm the importance of ascending relays in the functional innervation of cortical networks at the macroscopic scale. Our observations of reduced modulation of this ascending relay in OCD is suggestive of a contextually induced thalamo-cortical deficit in network function. What might the impact of such a deficit be? Before addressing this question, we briefly review extant understanding of the functional role of thalamo-cortical relays.

Both ascending and descending thalamo-cortical relays are necessary to balance the flow of *ascending* sensory inputs to the cortex against the *descending* modulation of these inputs. Ascending relays regulate the flow of sensory information to the cortex, whereas descending relays (dPFC → Thal) can directly excite or inhibit thalamic relay neurons via GABAergic neurons of the thalamic reticular nucleus (TRN), thus dynamically shaping the ascending inputs (121–123). The TRN provides inhibitory innervation to fellow thalamic nuclei while modulating the flow of sensory information to the cortex, as well as “influencing ongoing cortical activity by modulating cortico-thalamo-cortical transmission” (124, 125). As early as 1984, Francis Crick hypothesized an attentional role for the TRN, suggesting “if the thalamus is the gateway to the cortex, the reticular complex might be described as the guardian of the gateway”(126). Later, lesion studies on the TRN have shown impairments in the cognitive domain of attention (127, 128). Moreover, attention modulates visual processing via a reciprocal relationship between increasing LGN responses and decreasing neuronal TRN responses (30), consistent with Crick’s proposals. Thus, a vast connectivity network and polysynaptic receptors allowing tonic and burst modes of firing (129), regulate how the intrinsic circuitry dynamically modifies cortico-thalamic synchronization. This synchronization in turn is seen as a major functional basis for multiple cognitive functions (130). Unsurprisingly, a loss of this functional synchronization, particularly between cortical, striatal and thalamic circuits has been proposed to underlie OCD (131, 132) and has been documented in studies of resting state and task-based functional connectivity (55, 133, 134). Moreover, numerous studies implicate a broad range of neurocognitive deficits in attention, working memory, inhibition, and executive functions deficits in OCD (135). These have been observed during attention-related task paradigms such as the Trail-Making and Stroop Tasks and measurements of attentional set shifting, reversal learning, and cued tasked switching (136, 137). OCD patients also show hypo-activity in the dPFC as well as other PFC structures such as the OFC during reversal learning and task switching paradigms (51, 62, 138). From these results, Ahmari & Rauch have suggested that OCD subjects performing tasks with changing conditions “display less recruitment of multiple PFC structures” compared to controls. Furthermore, studies using the Tower of London task, a test which assesses goal-directed planning have also found hypo activation and slowed reaction times of the dPFC, suggesting a decrease in “dorsal prefrontal-striatal responsiveness” (138, 139).

Evidence of a loss of contextual modulation of the ascending relay falls firmly within this emerging framework. As we have noted, effective connectivity methods provide particular insight on the “causative” bases of inter-network effects (4, 9). They offer the possibility of identifying task-induced specificity in evoked network deficits. Such evidence is informative about how disease-related *traits* are manifested in functional brain states that are themselves transient and frequently task specific (91, 140). Such an approach may illuminate understanding of the mechanistic basis for pathology in neuropsychiatric conditions (2). We suggest that the reduced modulation of the ascending relay regardless of attention demand (Figure 7) reflects a basic loss of network tone evoked by sustained attention (22). It is plausible that this deficit reflects a relative loss of cortical innervation in OCD that subsequently impairs CST function (56), and that it provides functional evidence for increasingly clear impairments of thalamic structure in OCD (141). While circuit hyper-activity may be a trait emergent aspect of OCD (142), this may impact state-related network interactions that underlie basic processes such as attention. Thus, these deficits may affect how sensory information propagates to the cortex, where maladaptive inputs impact the fidelity of cortical network function in the disorder. This loss of network fidelity leads to a degradation of internal signals in the OCD brain, results in the emergence of erroneous perceptions (“something is wrong”), and eventually drives the anxieties that are characteristic of the disorder (143) and that drive trait-related hyperactivity.

The current study was partly motivated by prior investigations on the contextual modulation of cortico-thalamic pathways by attention demand (22), and which utilized a similar approach albeit only in healthy adolescents. That study used a winner-take-all approach from Bayesian model selection (26, 41), subsequently using Bayesian Parameter Averaging to estimate connectivity parameters from the likeliest generative model architecture. By comparison, BMA as employed in the current investigation is used to weight connectivity parameter estimates *across all models*, based on each model’s posterior probability. BMA is suitable for estimating parameters values for large model spaces (88), avoids uncertainties of model selection and mitigates any brittleness associated with parameter estimation (144). These differences in DCM-related methodology, as well as the focus on inter-group comparisons (as opposed to intra-group effects) may account for some differences between the sets of results from the two studies. For instance, a critical difference was that Jagtap and Diwadkar (22) noted differential effects of demand on the contextual modulation of the *descending* dPFC → Thal pathway (High Demand > Low Demand), suggestive of the role of frontally driven attention gain on the thalamus when demand for attention-resources was high. This effect was not replicated in the current study (see Figure 5), though our results on the ascending relay (significant contextual modulation for both low and high attention demand) in healthy controls (Figures 5, 7) was consistent with observations from the previous work.

Effective connectivity studies are beginning to illuminate understanding about *casual* brain network interactions in OCD. For example, using a reward learning task, Alves-Pinto et al., showed alterations in intrinsic connectivity and contextual modulation of fronto-striatal circuits, mainly from the ventromedial PFC (vmPFC) to the left and right orbitofrontal cortex (OFC) (145). In another study, Schlösser and colleagues used a Stroop color-word task to elicit aberrant effective connectivity pathways between OCD and HC in the fronto-cingulate system, consistent with an over-active error control system during decision-making observed in OCD (13). Han et al., showed that negative emotional distraction (working memory task) induced significantly reduced contextual modulation in the dorsolateral PFC (DLPFC) to OFC pathway in OCD compared to HC (146). Finally, van Velzen and colleagues have documented altered directionality of the connectivity from the amygdala to the OFC in OCD participants during a stop signal task (12).

### Limitations and conclusion

Understanding the relationship between behavior and brain network dynamics is perhaps *the* central challenge in human neuroscience (147) and the thalamo-cortical network appears to play a central role in mediating this relationship (148). Our focus on thalamo-cortical networks in OCD (1) reflects understandable interest in the role of the thalamus in the disorder (149), and (2) acknowledges the value of methods like DCM in revealing task-induced “mechanistic” deficits in brain networks (68). However, several limitations to our intriguing results should be acknowledged. Firstly, task-based fMRI results provide crucial information about demand-induced modulation of network connectivity and its relevance for psychiatric conditions in ways that resting state studies cannot (10). However, understanding specifically how impairments in micro or macro-circuits in the brain *explain* mental disorders remains a significant open problem in clinical neuroscience (150) and one that limits interpretations from a majority of fMRI studies including our own. Secondly, an absence of longitudinal data limits our ability to comment on the long-term impact of our observed connectivity deficits on the course of OCD symptomatology. Finally, as an exploration on putative mechanisms (see Introduction), we are not in a position to comment on whether treatment (or time) results in an amelioration of our observed connectivity impairments. This brief list of limitations is far from exhaustive, but serves as a road map for potential future investigations in the clinical neuroscience of the disorder.

In conclusion, large scale meta-analyses of *network function* are challenging given that network interactions are contextually tuned by task characteristics (151). However, we suggest that a growing corpus of studies on the effective (dys)connectivity of brain networks in OCD can contribute to understanding of the task conditions under which network dysfunction is evoked in the illness. Moreover, mechanism-based markers of dysfunction can also serve as therapeutic targets for long term intervention.

## Data availability statement

The original contributions presented in this study are included in the article/supplementary material, further inquiries can be directed to the corresponding author.

## Ethics statement

The studies involving human participants were reviewed and approved by Wayne State University IRB. Written informed consent to participate in this study was provided by the participants or their legal guardian/next of kin.

## Author contributions

MY conducted the analyses, created the figures, and wrote the manuscript. AC assisted in analyses and statistical methods. PE assisted in data collection and clinical analyses. GH assisted in clinical characterization and data collection. DR assisted in clinical characterization, data collection, and study design. VD oversaw all aspects of the analyses, writing, and presentation. All authors contributed to the article and approved the submitted version.

## Footnotes

1
https://www.fil.ion.ucl.ac.uk/spm/
